# Supplementary material for: Clinical utility of polygenic scores for cardiometabolic disease in Arabs
Source: Nat Commun. 2023 Oct 18;14:6535. doi: 10.1038/s41467-023-41985-1 (PMC10584889; doi:10.1038/s41467-023-41985-1)
Supplement: Supplementary file 1 — Supplementary Information [file 41467_2023_41985_MOESM1_ESM.pdf]

Clinical utility of polygenic scores for cardiometabolic disease in Arabs

Shim I et al.

Supplementary Information

Table of Contents

Supplementary Table 1. List of scoring files for polygenic scores from the PGS catalog

Supplementary Table 2. Results of associations between published polygenic scores and cardiometabolic traits in Arabs vs. Europeans

Supplementary Table 3. List of summary statistics used for polygenic score calculation

Supplementary Table 4. List of polygenic score methods used with a brief description and parameters

Supplementary Table 5. Characteristics of training and validation datasets

Supplementary Table 6. Comparison of multiple scores for binary diseases in the training set

Supplementary Table 7. Comparison of multiple scores for continuous traits in the training set

Supplementary Table 8. The interplay of coronary artery disease polygenic score with conventional risk factors

Supplementary Table 9. Characteristics of coronary artery disease patients with high (top quintile) vs. low (bottom quintile) polygenic risk

Supplementary Table 10. Characteristics of type 2 diabetes patients with high (top quintile) vs. low (bottom quintile) polygenic risk

Supplementary Table 11. Characteristics of Arab-matched vs. European-ancestry participants in the UK Biobank

Supplementary Figure 1. Principal component analysis comparison of Arab case-control cohort (N=5,399) and Arab reference population (N=1,017)

Supplementary Figure 2. Performance of polygenic scores in Arabs by imputation panel used

Supplementary Figure 3. Raw distribution of Arab-optimized polygenic scores in reference population, cases, and controls

Supplementary Figure 4. Stratification of disease risk or measured trait by polygenic score

Supplementary Figure 5. Performance of type 2 diabetes polygenic score in different patient subgroups

Supplementary Figure 6. Selection of an Arab-matched group from the UK Biobank dataset

Supplementary Figure 7. Comparison of association performance between the Arab-matched and European ancestry cohorts in the UK Biobank dataset

Supplementary Table 1. List of scoring files for polygenic scores from the PGS catalog

Trait	PGS catalog ID	Publication	Derivation Strategy	Tuning Parameter	No. variants available	Source of variant associations (GWAS)
Coronary artery disease	PGS000013	Khera AV et al. Nat Genet (2018)[1]	LDpred	ρ = 0.001	6,630,150	CARDIoGRAMplusC4D
Type 2 diabetes	PGS000014	Khera AV et al. Nat Genet (2018)[1]	LDpred	ρ = 0.01	6,917,436	DIAGRAM, EPIC, GERA
Cardiomyopathy	PGS002051	Privé F et al. Am J Hum Genet (2022)[2]	LDpred2	auto	642,241	UKB
LDL cholesterol	PGS000892	Graham SE et al. Nature (2021)[3]	PRS-CS	auto	1,239,184	GLGC
HDL cholesterol	PGS002781	Kanoni S et al. Genome Biol (2022)[4]	PRS-CS	auto	1,239,184	GLGC
Triglycerides	PGS002784	Kanoni S et al. Genome Biol (2022)[4]	Pruning and Thresholding	r2=0.1, p-value=5e-3, 500kb	30,071	GLGC
Systolic blood pressure	PGS002238	Breeyear JH et al. Circ Genom Precis Med (2022)[5]	PRS-CS after P-value thresholding	p-value=1e-1	1,119,444	MVP, BBJ, UKB
Diastolic blood pressure	PGS002239	Breeyear JH et al. Circ Genom Precis Med (2022)[5]	PRS-CS	p-value=1e-1	1,119,054	MVP, BBJ, UKB
Body mass index	PGS000027	Khera AV et al. Cell (2019)[6]	LDpred	ρ = 0.03	2,100,302	GIANT
Height	PGS002804	Yengo L et al. Nature (2022)[7]	SBayesC	-	1,103,042	GIANT

To compare the performance of the scores in Arabs from this study vs. European-ancestry participants from the UK Biobank, previously developed and published polygenic scores for 10 cardiometabolic diseases and traits were downloaded from the PGS catalog (https://www.pgscatalog.org/).

Supplementary Table 2. Results of associations between published polygenic scores and cardiometabolic traits in Arabs vs. European-ancestry participants in the UK Biobank

Trait	Score	Arabs in the study population					European-ancestry participants in the UK biobank				
**Categorical traits**	**PGS Catalog ID**	**N Total**	**N Cases (%)**	**N variants in PRS**	**OR per SD (95% CI)**	**AUC (95% CI)**	**N Total**	**N Cases (%)**	**N variants in PRS**	**OR per SD (95% CI)**	**AUC (95% CI)**
Coronary artery disease	PGS000013	2,682	1,712 (63.8)	5,706,928	1.41 (1.31-1.5)	0.7909 (0.7726-0.8091)	2,682	1,760 (65.6)	6,353,896	1.78 (1.69-1.88)	0.7944 (0.7760-0.8128)
Type 2 diabetes	PGS000014	2,673	1,468 (54.9)	5,786,938	1.41 (1.32-1.49)	0.7054 (0.6854-0.7255)	2,673	1,513 (56.6)	6,572,678	1.82 (1.73-1.91)	0.7325 (0.7132-0.7518)
Cardiomyopathy	PGS002051	2,590	235 (9.1)	621,802	1.01 (0.88-1.16)	0.6277 (0.589-0.6665)	2,590	234 (9.0)	623,878	3.13 (2.98-3.27)	0.8154 (0.7826-0.8483)
**Continuous traits**	**PGS Catalog ID**	**N Total**	**Mean (SD)**	**N variants in PRS**	**Effect size (SE)**	**Adjusted R-sq**	**N Total**	**Mean (SD)**	**N variants in PRS**	**Effect size (SE)**	**Adjusted R-sq**
LDL Cholesterol [mg/dL]	PGS000892	2,201	134.97 (52.61)	1,068,974	10.06 (1.10)	0.0405	2,201	133.43 (42.49)	1,077,905	17.3 (0.78)	0.2829
HDL Cholesterol [mg/dL]	PGS002781	2,208	45.08 (13.46)	1,112,500	3.49 (0.27)	0.1351	2,208	46.45 (13.87)	1,084,067	6.12 (0.26)	0.2469
Triglyceride [mg/dL]	PGS002784	2,212	165.05 (113.75)	12,709	25.19 (2.34)	0.0682	2,212	192.79 (140.65)	16,937	44.85 (2.81)	0.2111
Systolic blood pressure [mmHg]	PGS002238	2,613	145.99 (24.02)	1,069,203	1.08 (0.45)	0.0961	2,613	151.36 (28.99)	1,078,143	0.57 (0.59)	0.039
Diastolic blood pressure [mmHg]	PGS002239	2,613	82.90 (13.62)	1,069,056	0.71 (0.26)	0.0249	2,613	84.66 (16.26)	1,077,813	1.57 (0.33)	0.0825
Body mass index [kg/m^2]	PGS000027	2,553	29.23 (6.02)	2,015,065	0.97 (0.11)	0.0790	2,553	29.63 (6.92)	2,035,054	2.22 (0.14)	0.1232
Height [m]	PGS002804	2,553	1.61 (0.094)	1,054,056	0.027 (0.0013)	0.5299	2,553	1.69 (0.10)	1,098,475	0.046 (0.0012)	0.7068

Using 1:1 matched cohorts for age, sex, and case-control ratio for categorical traits, or mean value for continuous traits from UK biobank, the performance of published scores was compared in Arabs vs. individuals of European ancestry. For assessing the association between the score and each disease outcome, a logistic regression model adjusted for age, sex, array version, and the first 10 PCs was utilized. Continuous traits were analyzed using linear regression models with the same covariates. (OR per SD: odd ratio per standard deviation, AUC: area under the receiver operating characteristic curve, CI: confidence interval, SE: standard error)

Supplementary Table 3. List of summary statistics used for polygenic score calculation

Trait	GWAS	Ancestry	Sample size	N Cases	N Controls	Publication
Coronary artery disease	CARDIoGRAM plusC4D+UKB	Mostly EUR ancestry	547,261	122,733	424,528	van der Harst P, et al. Circ Res (2018)[8]
Type 2 diabetes	DIAGRAM	Multi-ancestry	1,339,889	180,834	1,159,055	Mahajan A, et al. Nat Genet (2022)[9]
Cardiomyopathy (left ventricular measurements on MRI)	UKB	Mostly EUR ancestry	36,041	NA	NA	Pirruccello J, et al. Nat Commun (2020)[10]
LDL cholesterol	QGP, GLGC (5 ancestries)	Multi-ancestry	QGP (Arabs): 5,972<br>GLGC (AFR): 99,432<br>GLGC (EAS): 146,492<br>GLGC (EUR): 1,320,016<br>GLGC (AMR): 48,057<br>GLGC (SAS): 40,963	NA	NA	Thareja G, et al. Nat Commun (2021)[11]<br>Graham SE, et al. Nature (2021)[3]
HDL cholesterol				NA	NA	
Triglycerides				NA	NA	
Systolic blood pressure	ICBP+UKB	EUR ancestry only	757,601	NA	NA	Evangelou E, et al. Nat Genet (2018)[12]
Diastolic blood pressure				NA	NA	
Body mass index	QGP, PAGE, BBJ, GIANT	Multi-ancestry	QGP (Arabs): 6,039<br>PAGE (AMR): 49,796<br>BBJ (EAS): 159,148<br>GIANT (EUR): 795,640	NA	NA	Thareja G, et al. Nat Commun (2021)[11]<br>Wojcik G, et al. Nature (2019)[13]<br>Sakaue S, et al., Nat Genet (2021)[14]<br>Yengo L, et al. Hum Mol Genet (2018)[15]
Height				NA	NA	

To compute polygenic scores, we obtained variant effect size from the largest and most diverse genome-wide association study (GWAS) available for each trait. (AFR: African; AMR: Admixed American; EAS: East Asian; EUR: European; SAS: South Asian)

Supplementary Table 4. List of polygenic score methods used with a brief description and parameters

Method	Software	Parameters	Description	LD reference	Publication
PRSice-2	PRSice-2	The default values for clumping parameters (--clump-kb 250kb \ --clump-p 1.0 \ --clump-r2 0.1) and 14 p-value thresholds (--bar-levels 1e-08,1e-07,1e-06,1e-05,3e-05,0.0001,0.0003,0.001,0.003,0.01,0.03,0.1,0.3,1)	Clumping and P-value thresholding (C+T)	In-sample LD matrix	Choi SW, et al. GigaScience (2019)[16]
PRS-CS	PRS-CS	5 global scaling parameter, phi = {auto, 1, 1e-2, 1e-4, 1e-6}, and the default values for hyper-parameters a=1 and b=0.5.	Bayesian shrinkage	LD reference panel constructed using 1000G phase3 EUR	Ge T, et al. Nat Commun (2019)[17]
PRS-CSx	PRS-CS	5 global scaling parameter, phi = {auto, 1, 1e-2, 1e-4, 1e-6}, and the default values for hyper-parameters a=1 and b=0.5.	Bayesian shrinkage	LD reference panel constructed using Arab samples and five super populations (AFR, AMR, EAS, EUR, SAS) in 1000G	Ruan Y, et al. Nat Genet (2022)[18]
LDpred2(-grid)	bigsnpr (R package)	51 combinations of heritability (h2) and non-zero effect fractions (p).<br>h2_seq <- round(h2_est * c(0.7, 1, 1.4) , 4)<br>p_seq <- signif(seq_log(1e-4, 1, length.out = 17), 2)<br>params <- expand.grid(p = p_seq, h2 = h2_seq, sparse = FALSE)	Bayesian shrinkage	LD reference panel constructed using 1000G phase3 EUR	Privé F, et al. Bioinformatics (2021)[19]
lassosum2	bigsnpr (R package)	Combinations of delta, a vector of shrinkage parameters to try (L2-regularization), and nlambda, the number of different lambdas to try (L1-regularization).<br>delta = signif(seq_log(0.001, 3, 6), 1), nlambda = 20	Lasso regression-based	LD reference panel constructed using 1000G phase3 EUR	Privé F, et al. HGG Advances (2022)[20]

Five currently available approaches used to derive Arab-optimized polygenic scores. (AFR: African; AMR: Admixed American; EAS: East Asian; EUR: European; SAS: South Asian)

Supplementary Table 5. Characteristics of training and validation datasets

Characteristic	Training dataset (N=2,700)	N total after excluding missing values	Validation dataset (N=2,699)	N total after excluding missing values
Sex, male (%)	1733 (64.2)	2,700	1740 (64.5)	2,699
Age, mean (SD)	54.95 (14.80)	2,700	54.72 (14.84)	2,699
Coronary artery disease, n (%)	1779 (66.2)	2,688	1712 (63.8)	2,682
Type 2 diabetes, n (%)	1511 (56.2)	2,687	1468 (54.9)	2,673
Cardiomyopathy, n (%)	244 (9.5)	2,581	235 (9.1)	2,590
LDL cholesterol[a], mean (SD) [mg/dL]	136.66 (54.13)	2,246	134.97 (52.61)	2,201
HDL cholesterol[b], mean (SD) [mg/dL]	45.32 (13.02)	2,251	45.08 (13.46)	2,208
Triglycerides[c], mean (SD) [mg/dL]	166.39 (110.03)	2,260	165.05 (113.75)	2,212
Systolic blood pressure[d], mean (SD) [mmHg]	147.23 (23.81)	2,606	145.99 (24.02)	2,613
Diastolic blood pressure[d], mean (SD) [mmHg]	83.21 (13.05)	2,606	82.90 (13.62)	2,613
Body mass index, mean (SD) [kg/m^2]	29.39 (6.08)	2,570	29.23 (6.02)	2,553
Height, mean (SD) [m]	1.61 (0.09)	2,568	1.61 (0.09)	2,553

The number of missing values varies across the characteristics. [a]LDL cholesterol levels were adjusted for statins, ezetimibe, and fibrate use. [b]HDL cholesterol levels were adjusted for fibrate use. [c]Triglycerides levels were adjusted for statin and fibrate use. [d]Systolic and diastolic blood pressures were adjusted for antihypertensive medication (ACE inhibitors, angiotensin receptor blockers, beta-blockers, calcium channel blockers, diuretics, and nitrates) use.

Supplementary Table 6. Comparison of multiple scores for binary diseases in the training set

Categorical traits	Derivation Strategy	Tuning Parameter	No. of variants in PRS	OR per SD (95% CI)	AUC (95% CI)	No. of available models
Coronary artery disease	PRSice-2	1.00E-05	834	1.35 (1.26-1.45)	0.7909 (0.7722-0.8096)	14
Coronary artery disease	PRS-CS	auto	1,066,917	1.38 (1.28-1.47)	0.7915 (0.7729-0.8102)	5
Coronary artery disease	LDpred2	P9_hsq0.7	1,008,661	1.38 (1.29-1.47)	0.7916 (0.7728-0.8103)	24
Coronary artery disease	lassosum2	s:0.6_nl:12	10,440	1.38 (1.29-1.48)	0.7922 (0.7736-0.8109)	104
Type 2 diabetes	PRSice-2	0.001	5,269	1.60 (1.51-1.69)	0.7237 (0.7041-0.7434)	14
Type 2 diabetes	PRS-CS	1.00E-04	1,068,166	1.85 (1.75-1.94)	0.7451 (0.7263-0.764)	5
Type 2 diabetes	LDpred2	P12_hsq0.7	1,008,959	1.83 (1.74-1.92)	0.7441 (0.7252-0.763)	18
Type 2 diabetes	lassosum2	s:3_nl:19	71,585	1.82 (1.73-1.91)	0.7433 (0.7244-0.7622)	107
Cardiomyopathy	PRSice-2	0.3	135,201	1.13 (1.00-1.27)	0.6365 (0.5987-0.6744)	14
Cardiomyopathy	PRS-CS	1.00E-06	1,069,402	1.41 (1.18-1.76)	0.6624 (0.6260-0.6988)	5
Cardiomyopathy	LDpred2	P7_hsq0.7	1,010,014	1.50 (1.24-1.90)	0.6719 (0.6355-0.7082)	51
Cardiomyopathy	lassosum2	s:0.6_nl:18	20,399	1.49 (1.24-1.87)	0.6706 (0.6338-0.7074)	113

For binary disease outcomes, the best model was determined by the score with the maximum area under the curve (AUC) in a logistic regression model adjusted for age, sex, array version, and the first 10 PCs. (OR per SD: odd ratio per standard deviation, AUC: area under the receiver operating characteristic curve, CI: confidence interval)

Supplementary Table 7. Comparison of multiple scores for continuous traits in the training set

Continuous traits	Derivation Strategy	Tuning Parameter	No. of variants in PRS	Effect size (SE)	Adjusted R-sq	No. of available models
LDL cholesterol	PRSice-2	3.00E-05	4,820	9.30 (1.13)	0.0310	14
LDL cholesterol	PRS-CS	auto	1,069,622	8.93 (1.13)	0.0288	5
LDL cholesterol	PRS-CSx	1.00E-04	-	11.04 (1.12)	0.0431	5
LDL cholesterol	LDpred2	P14_hsq0.7	1,010,014	9.35 (1.13)	0.0314	9
LDL cholesterol	lassosum2	s:0.001_nl:20	21,678	10.34 (1.12)	0.038	92
HDL cholesterol	PRSice-2	0.0001	8,651	2.33 (0.26)	0.0877	14
HDL cholesterol	PRS-CS	auto	1,068,166	3.18 (0.26)	0.1155	5
HDL cholesterol	PRS-CSx	1.00E-04	-	3.51 (0.26)	0.1287	5
HDL cholesterol	LDpred2	P16_hsq0.7	1,010,014	3.30 (0.26)	0.1203	3
HDL cholesterol	lassosum2	s:3_nl:20	145,512	3.44 (0.26)	0.1256	88
Triglycerides	PRSice-2	1.00E-05	4,981	22.93 (2.25)	0.0568	14
Triglycerides	PRS-CS	1.00E-04	1,069,677	26.31 (2.23)	0.0706	5
Triglycerides	PRS-CSx	1.00E-06	-	31.36 (2.20)	0.0948	5
Triglycerides	LDpred2	P15_hsq0.7	1,010,014	27.32 (2.23)	0.0751	6
Triglycerides	lassosum2	s:0.005_nl:20	54,623	31.53 (2.20)	0.0956	87
Systolic blood pressure	PRSice-2	0.0003	7,743	1.58 (0.44)	0.0916	14
Systolic blood pressure	PRS-CS	auto	1,056,790	2.73 (0.44)	0.1003	5
Systolic blood pressure	LDpred2	P14_hsq0.7	1,000,653	2.46 (0.44)	0.0978	14
Systolic blood pressure	lassosum2	s:0.6_nl:19	253,740	2.56 (0.44)	0.0987	108
Diastolic blood pressure	PRSice-2	1.00E-08	1,569	1.09 (0.25)	0.0318	14
Diastolic blood pressure	PRS-CS	1.00E-04	1,058,103	1.87 (0.25)	0.0454	5
Diastolic blood pressure	LDpred2	P13_hsq0.7	1,001,871	1.63 (0.25)	0.0403	12
Diastolic blood pressure	lassosum2	s:3_nl:15	25,857	1.95 (0.25)	0.0471	108
Body mass index	PRSice-2	0.01	22,674	0.99 (0.11)	0.1068	14
Body mass index	PRS-CS	1.00E-02	922,653	1.10 (0.11)	0.1128	5
Body mass index	PRS-CSx	auto	-	1.27 (0.11)	0.1243	5
Body mass index	LDpred2	P14_hsq0.7	876,537	1.02 (0.11)	0.1083	11
Body mass index	lassosum2	s:0.001_nl:20	214,522	1.10 (0.11)	0.1128	112
Height	PRSice-2	0.03	43,357	0.022 (0.0013)	0.4832	14
Height	PRS-CS	auto	921,738	0.025 (0.0013)	0.4993	5

Height	PRS-CSx	auto	-	0.025 (0.0013)	0.5040	5
Height	LDpred2	P17_hsq0.7	875,698	0.022 (0.0013)	0.4878	1
Height	lassosum2	s:3_nl:19	147,234	0.024 (0.0013)	0.4967	103

For continuous traits, the best model was determined by the score with the maximum adjusted R^2 in a linear regression model adjusted for age, sex, array version, and the first 10 PCs.

Supplementary Table 8. The interplay of coronary artery disease polygenic score with conventional risk factors

Model	OR per SD	Lower 95% CI	Upper 95% CI	P-value	P-value for interaction
CAD ~ PRS + age + sex + batch + 10 PCs (baseline)	1.51	1.42	1.61	7.21E-18	
CAD ~ PRS + age + sex + batch + 10 PCs + Smoking + PRS*Smoking	1.50	1.38	1.62	3.82E-11	0.95
CAD ~ PRS + age + sex + batch + 10 PCs + Obesity + PRS*Obesity	1.46	1.33	1.59	8.69E-09	0.43
CAD ~ PRS + age + sex + batch + 10 PCs + SBP + PRS*SBP	1.33	0.72	1.94	3.60E-01	0.65
CAD ~ PRS + age + sex + batch + 10 PCs + DM + PRS*DM	1.39	1.25	1.52	1.97E-06	0.12
CAD ~ PRS + age + sex + batch + 10 PCs + LDL + PRS*LDL	1.70	1.41	1.99	3.62E-04	0.38

In the validation dataset, adjusting for smoking, obesity, systolic blood pressure (SBP), type 2 diabetes (DM), and LDL cholesterol (LDL) had no significant effect on the relationship between polygenic score and coronary artery disease (CAD). There was also no interaction between these risk factors and the polygenic score for CAD. The odds ratios were assessed using a logistic regression model that included age, sex, array version, the first 10 principal components of ancestry, each conventional risk factor, and its interaction with the polygenic score as covariates. P-values were determined using a two-sided Wald test. (OR per SD: odd ratio per standard deviation, CI: confidence interval)

Supplementary Table 9. Characteristics of coronary artery disease patients with high (top quintile) vs. low (bottom quintile) polygenic risk

Characteristic	Low polygenic risk (Bottom quintile group)	High polygenic risk (Top quintile group)	P-value
N	441	1042	
Sex, male (%)	325 (73.7)	770 (73.9)	0.988
Age, mean (SD)	60.30 (12.53)	58.05 (11.55)	0.001
Current smoking, n (%)	190 (43.3)	497 (47.8)	0.122
Obesity, n (%)	185 (44.3)	406 (41.1)	0.305
Hypertension, n (%)	351 (81.4)	853 (85.1)	0.095
Type 2 diabetes, n (%)	294 (67.0)	704 (67.6)	0.872
Hypercholesterolemia, n (%)	150 (40.0)	416 (46.7)	0.032

Comparing the conventional risk factors of coronary artery disease patients with high (top quintile) polygenic risk to those with low (bottom quintile) polygenic risk. P-values were determined using Welch’s or Student’s two sample t-test. (SD: standard deviation)

Supplementary Table 10 Characteristics of type 2 diabetes patients with high (top quintile) vs. low (bottom quintile) polygenic risk

Characteristic	Low polygenic risk (Bottom quintile group)	High polygenic risk (Top quintile group)	P-value
N	321	1008	
Sex, male (%)	197 (61.4)	666 (66.1)	0.142
Age, mean (SD)	61.02 (11.60)	58.46 (11.39)	0.001
Current smoking, n (%)	120 (37.5)	403 (40.2)	0.431
Obesity, n (%)	166 (54.4)	422 (43.9)	0.002
Hypertension, n (%)	264 (84.6)	845 (86.4)	0.486
Coronary artery disease, n (%)	246 (76.9)	789 (78.4)	0.633
Hypercholesterolemia, n (%)	114 (41.6)	367 (42.2)	0.91

Comparing the conventional risk factors of type 2 diabetes patients with high (top quintile) polygenic risk to those with low (bottom quintile) polygenic risk. P-values were determined using Welch’s or Student’s two sample t-test. (SD: standard deviation)

Supplementary Table 11. Characteristics of Arab-matched vs. European-ancestry participants in the UK Biobank

Characteristic	Arab-matched (N=420)	European ancestry (N=223,901)
Sex, male (%)	66.9	47.15
Age, mean (SD)	52.40 (7.98)	56.86 (7.98)
LDL cholesterol, mean (SD) [mg/dL]	142.42 (35.29)	143.01 (33.14)
HDL cholesterol, mean (SD) [mg/dL]	46.73 (11.50)	56.29 (14.84)
Triglycerides, mean (SD) [mg/dL]	182.52 (121.92)	160.31 (95.31)
Systolic blood pressure, mean (SD) [mmHg]	133.69 (18.07)	141.66 (20.83)
Diastolic blood pressure, mean (SD) [mmHg]	81.30 (10.56)	83.28 (11.45)
Body mass index, mean (SD) [kg/m^2]	28.77 (4.32)	27.35 (4.72)
Height, mean (SD) [m]	1.68 (0.086)	1.69 (0.09)

SD: standard deviation

Supplementary Figure 1. Principal component analysis comparison of Arab case-control cohort (N=5,399) and Arab reference population (N=1,017)

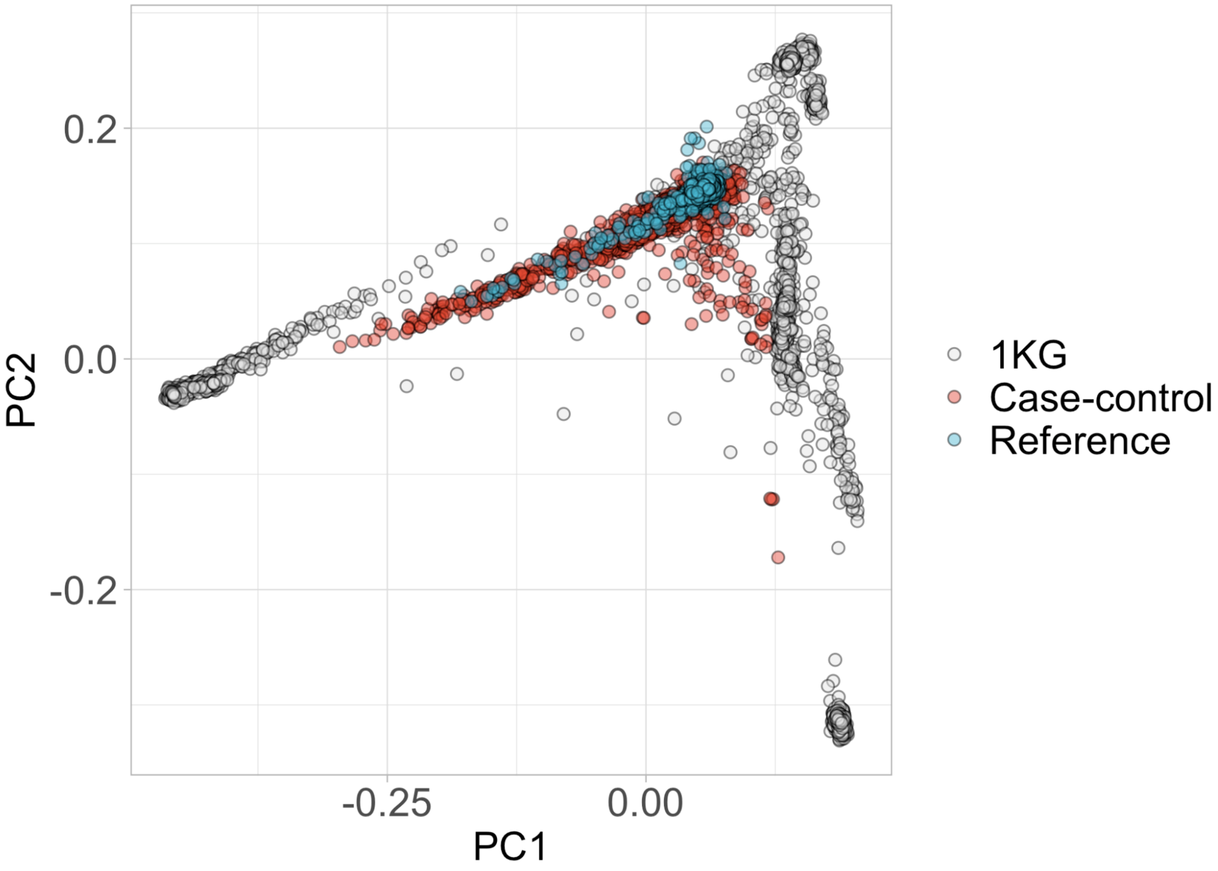

Principal components of ancestry plot showing that the reference population had similar genetic ancestry to the case-control study population.

Supplementary Figure 2. Performance of polygenic scores in Arabs by imputation panel used

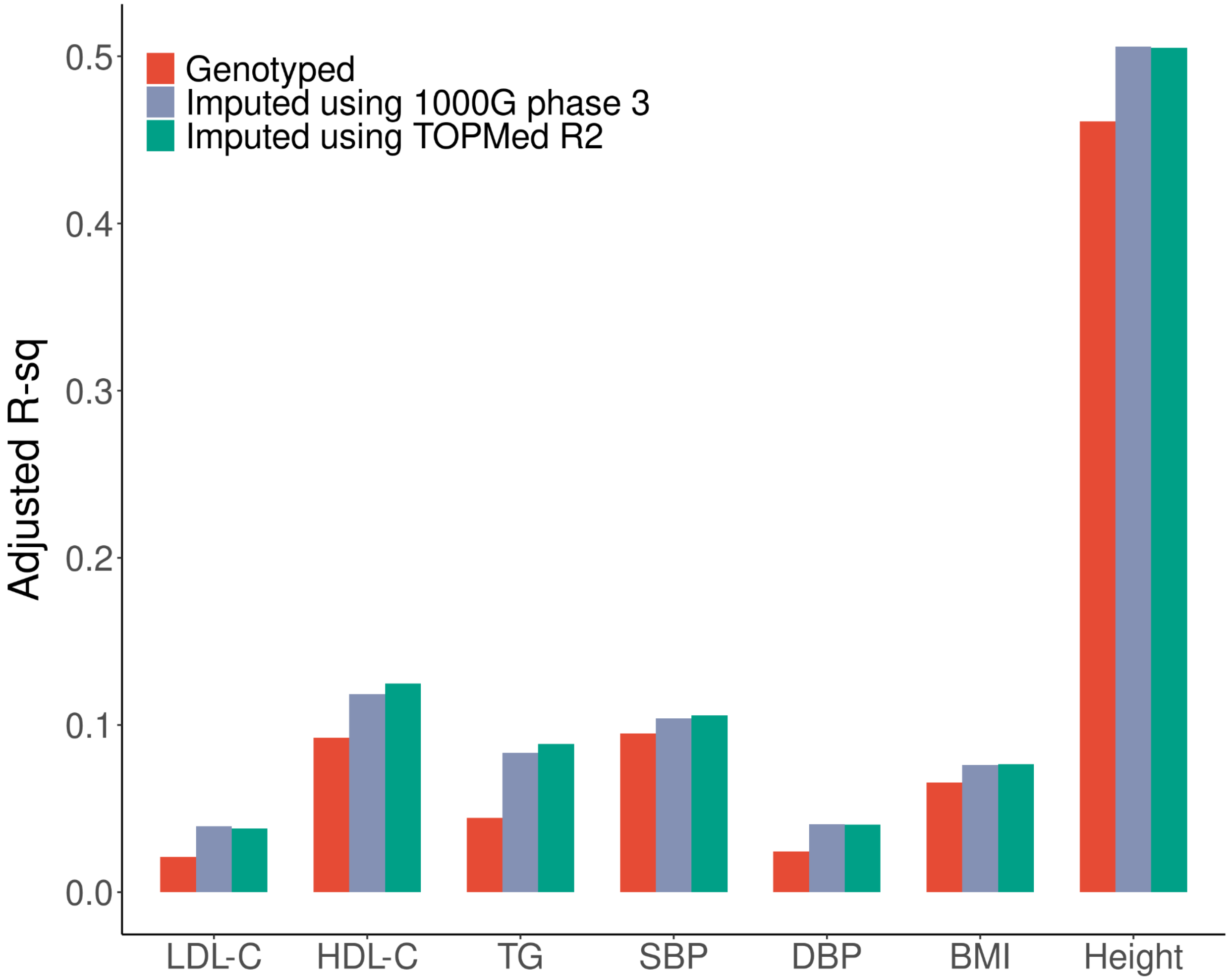

The performance of polygenic scores obtained from genotyping array data without imputation, imputed data using 1000G phase 3, and imputed data using TOPMed R2 were compared

Supplementary Figure 3. Raw distribution of Arab-optimized polygenic scores in reference population, cases, and controls

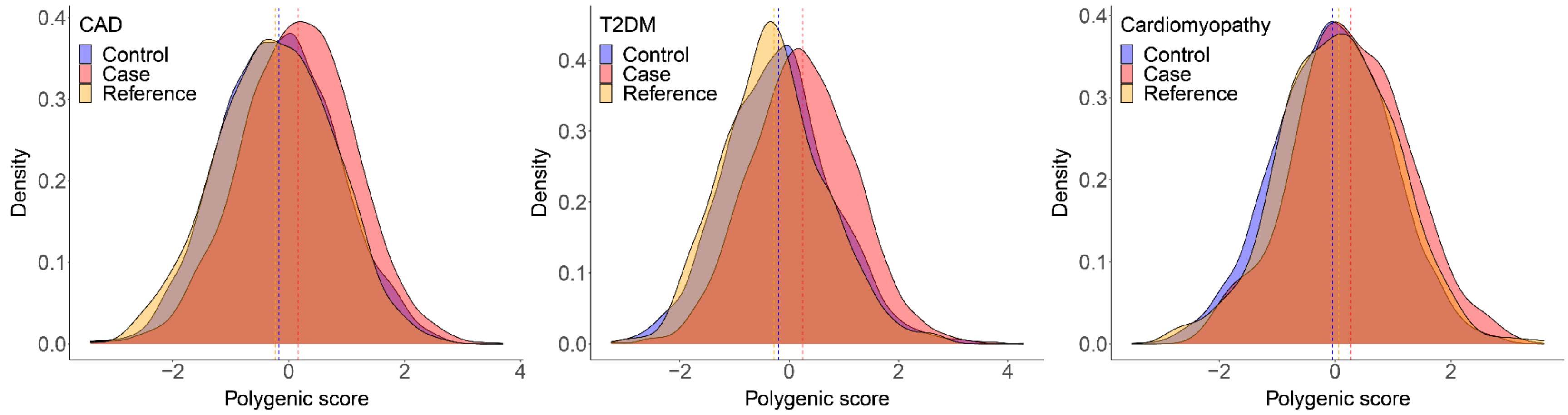

According to the density plots for coronary artery disease (CAD), type 2 diabetes (T2DM), and cardiomyopathy, the distribution of polygenic scores in the reference population was comparable to that of the control group.

Supplementary Figure 4. Stratification of disease risk or measured trait by polygenic score

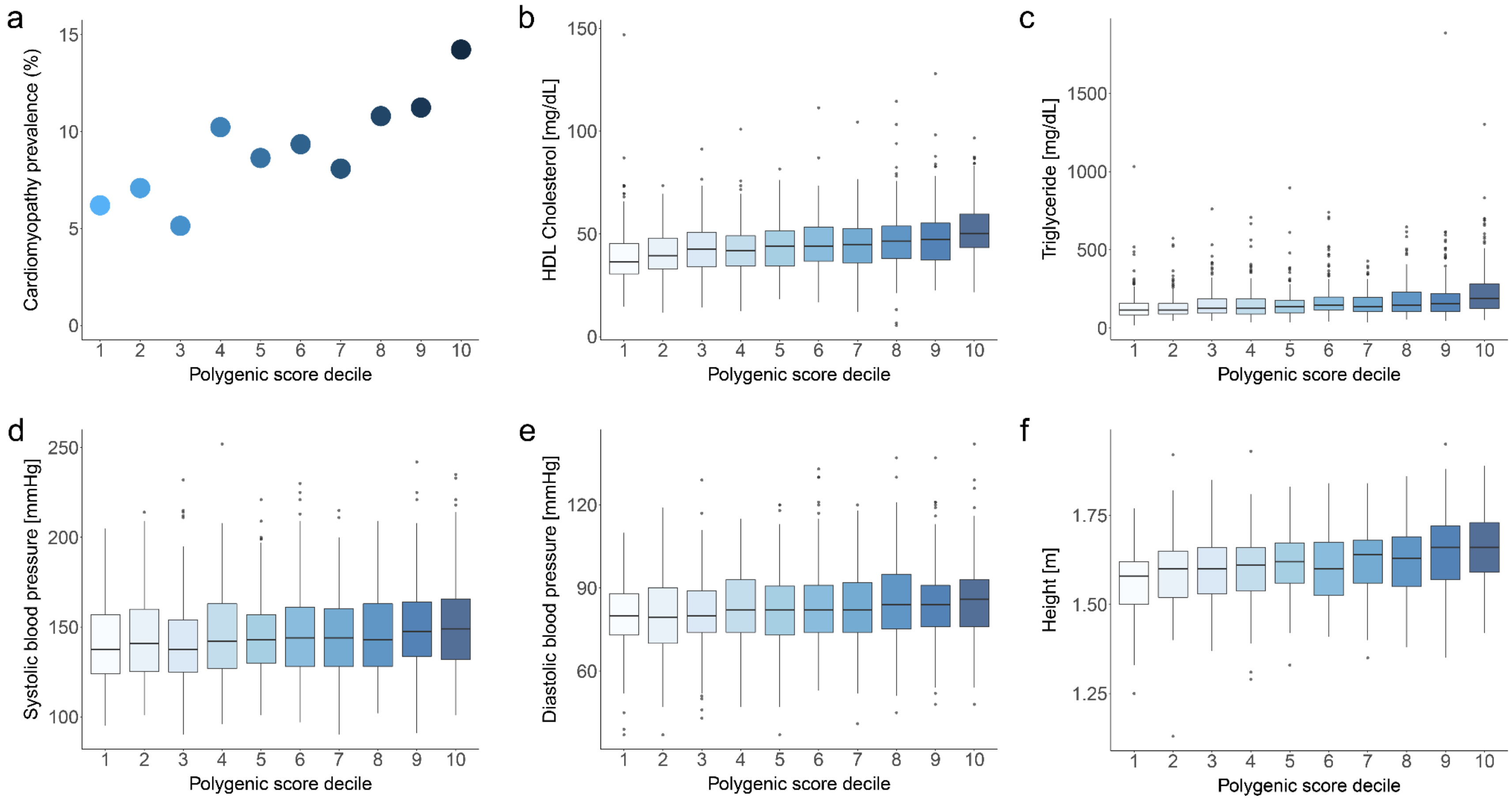

In the validation dataset, stratification according to polygenic score decile groups represented by dot plots of prevalence rates for binary disease and box plots for continuous traits.

Supplementary Figure 5. Performance of type 2 diabetes polygenic score in different patient subgroups.

		N Total	N Cases	OR (95% CI)	P-value
Type 2 diabetes (T2DM)		2,673	1,468	1.83 (1.74-1.92)	4.51×10^{-39}
Age	> 55 years	1,366	930	1.72 (1.59-1.84)	1.84×10^{-17}
	≤ 55 years	1,307	538	1.97 (1.83-2.11)	1.81×10^{-21}
Sex	Female	947	490	1.65 (1.49-1.80)	8.74×10^{-11}
	Male	1,726	978	1.93 (1.82-2.04)	1.04×10^{-29}
Obesity	Nonobese	1,503	752	1.95 (1.82-2.07)	1.85×10^{-25}
	Obese	1,028	640	1.81 (1.67-1.96)	2.38×10^{-15}

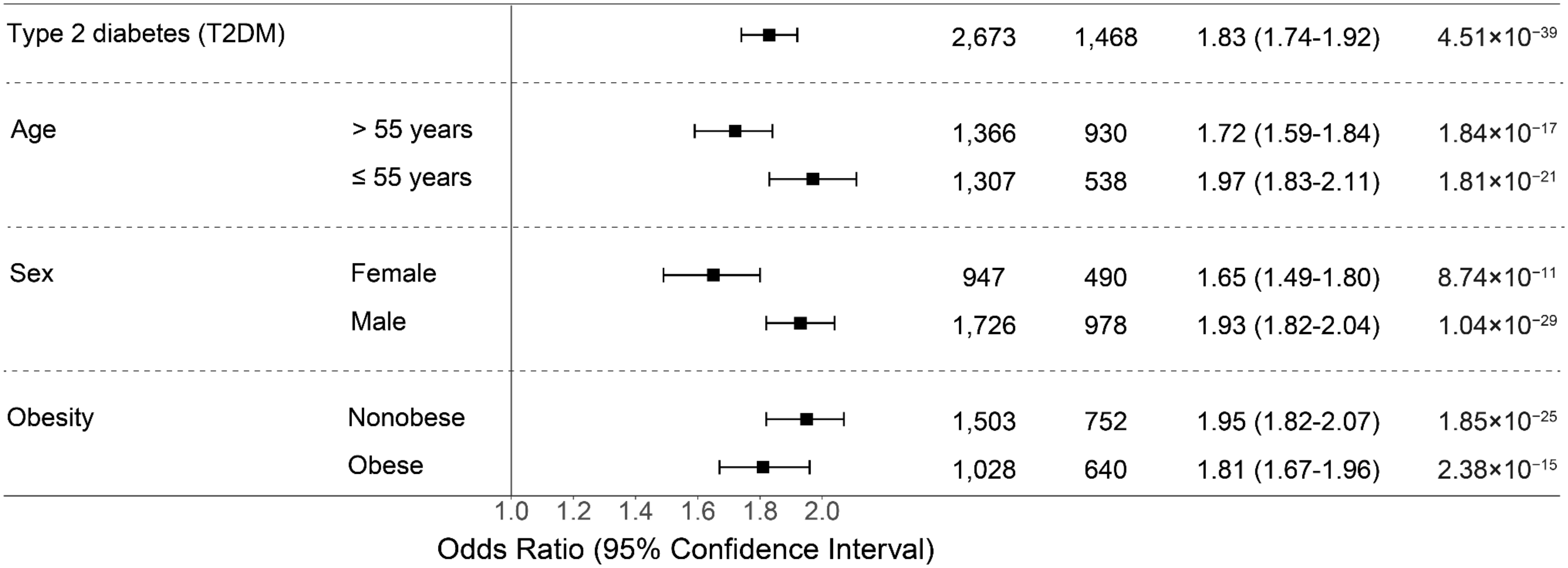

In the validation dataset, the performance of the polygenic score for type 2 diabetes in different patient subgroups by age, sex, and obesity was assessed. Obesity was defined as body mass index ≥ 30 kg/m2. The odds ratio was assessed in a logistic regression model with age, sex, array version, and the first 10 principal components of ancestry as covariates. (OR: odd ratio, CI: confidence interval)

Supplementary Figure 6. Selection of an Arab-matched group from the UK Biobank dataset

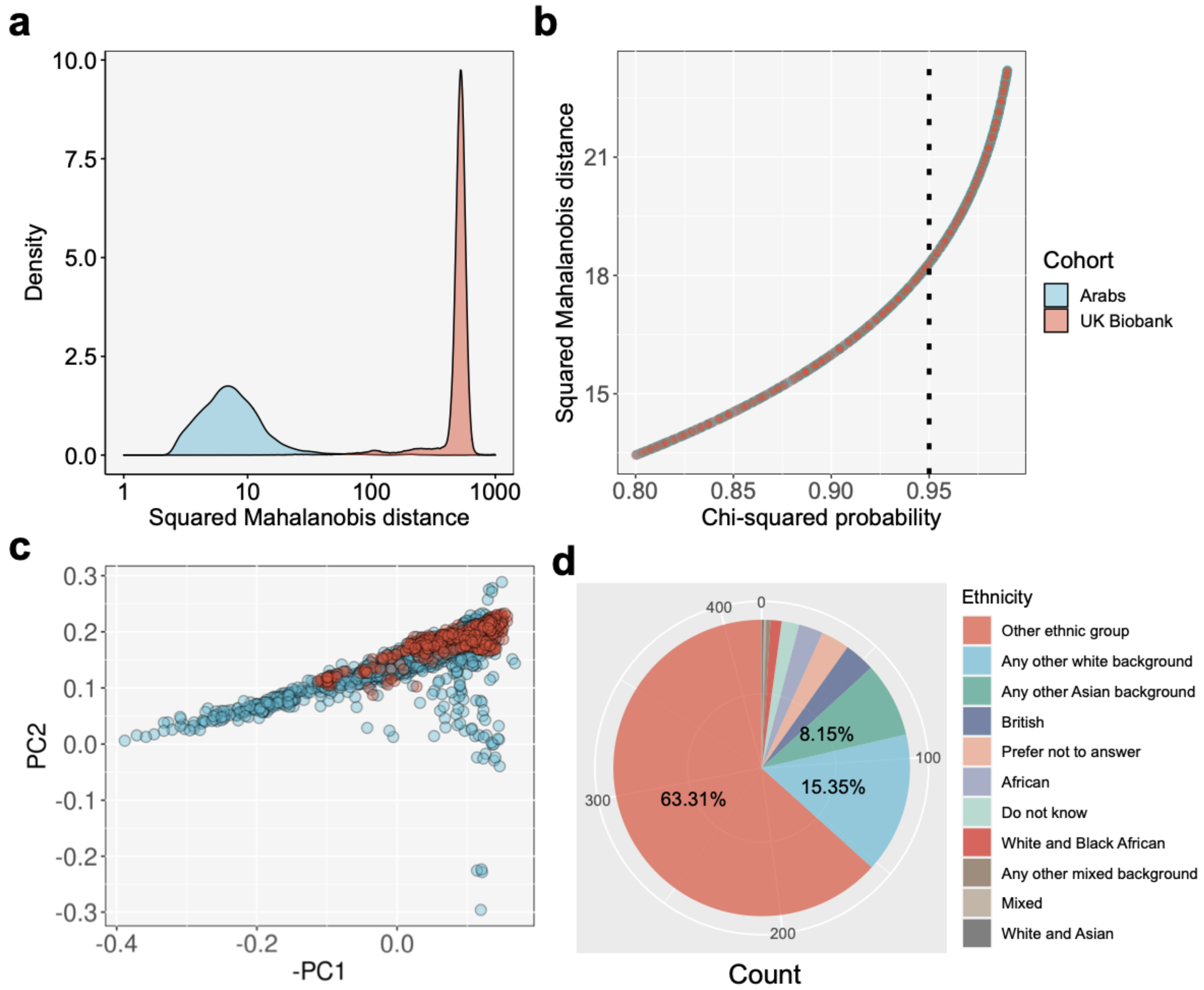

a. Distributions of Squared Mahalanobis distances from the centroid of unrelated Arab samples (N=5,884). The Mahalanobis distance was computed using the top 10 principal components (PC) of ancestry of the Arab and UK Biobank samples projected onto the 1000 Genomes PC space. **b.** A Q-Q plot of the squared Mahalanobis distance against the corresponding chi-square quantiles with 10 degrees of freedom. The dotted line indicates the 95th percentile, which was used as the cutoff value to select the Arab-matched group (N=420). **c.** A PC ancestry biplot showing Arab-matched participants from the UK Biobank projected onto the 1000 Genomes PC space. **d.** Pie chart showing the breakdown of the self-reported ethnicity of UK Biobank participants identified as Arab-matched (N=420).

Supplementary Figure 7. Comparison of association performance between the Arab-matched and European-ancestry cohorts in the UK Biobank dataset

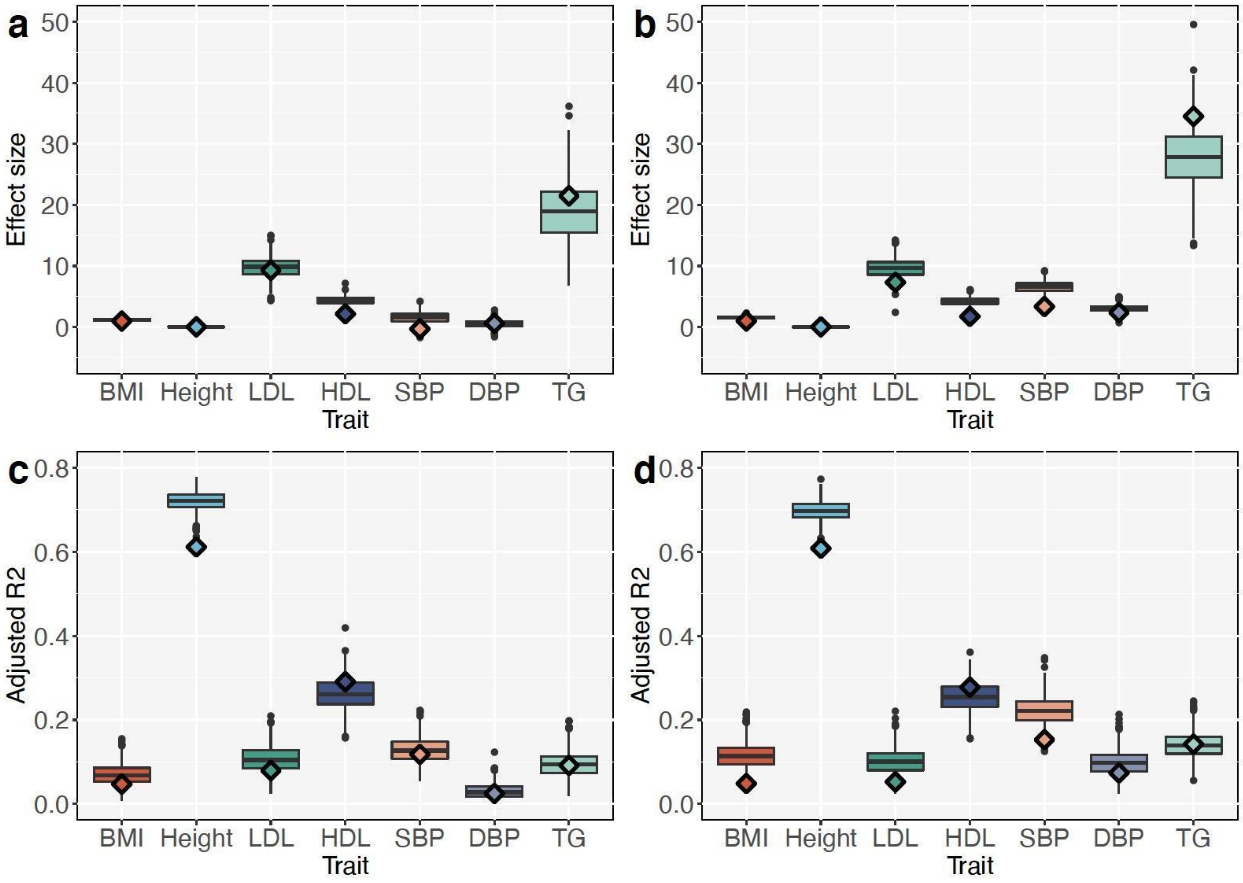

From 223,901 European-ancestry participants in the UK Biobank, 420 samples were randomly drawn 1,000 times to generate 1,000 groups, and their distribution of association measures (boxplot) were compared against the association measure from the 420 Arab-matched participants (diamond) for each of the 7 continuous traits. **a.** Comparative performance in effect size for published European-derived scores (Supplementary Table 1). **b.** Comparative performance in effect size for the Arab-optimized scores in this study (Table 2). **c.** Comparative performance in adjusted R^2 for published European-derived scores (Supplementary Table 1). **d.** Comparative performance in adjusted R^2 for Arab-optimized scores in this study (Table 2). Associations were derived using linear regression models adjusted for age, sex, array version, and the first 10 PCs.

Supplementary References

1. Khera, A. V. *et al.* Genome-wide polygenic scores for common diseases identify individuals with risk equivalent to monogenic mutations. *Nat. Genet.* **50**, 1219–1224 (2018).
2. Privé, F. *et al.* Portability of 245 polygenic scores when derived from the UK Biobank and applied to 9 ancestry groups from the same cohort. *Am. J. Hum. Genet.* **109**, 12–23 (2022).
3. Graham, S. E. *et al.* The power of genetic diversity in genome-wide association studies of lipids. *Nature* **600**, 675–679 (2021).
4. Kanoni, S. *et al.* Implicating genes, pleiotropy, and sexual dimorphism at blood lipid loci through multi-ancestry meta-analysis. *Genome Biol.* **23**, 268 (2022).
5. Breeyear, J. H., Shuey, M. M., Edwards, T. L. & Hellwege, J. N. Blood Pressure Polygenic Scores Are Associated With Apparent Treatment-Resistant Hypertension. *Circ. Genomic Precis. Med.* **15**, e003554 (2022).
6. Khera, A. V. *et al.* Polygenic Prediction of Weight and Obesity Trajectories from Birth to Adulthood. *Cell* **177**, 587-596.e9 (2019).
7. Yengo, L. *et al.* A saturated map of common genetic variants associated with human height. *Nature* **610**, 704–712 (2022).
8. van der Harst, P. & Verweij, N. Identification of 64 Novel Genetic Loci Provides an Expanded View on the Genetic Architecture of Coronary Artery Disease. *Circ. Res.* **122**, 433–443 (2018).
9. Mahajan, A. *et al.* Multi-ancestry genetic study of type 2 diabetes highlights the power of diverse populations for discovery and translation. *Nat. Genet.* **54**, 560–572 (2022).
10. Pirruccello, J. P. *et al.* Analysis of cardiac magnetic resonance imaging in 36,000 individuals yields genetic insights into dilated cardiomyopathy. *Nat. Commun.* **11**, 2254 (2020).
11. Thareja, G. *et al.* Whole genome sequencing in the Middle Eastern Qatari population identifies genetic associations with 45 clinically relevant traits. *Nat. Commun.* **12**, 1250

(2021).

12. Evangelou, E. *et al.* Genetic analysis of over 1 million people identifies 535 new loci associated with blood pressure traits. *Nat. Genet.* **50**, 1412–1425 (2018).

13. Wojcik, G. L. *et al.* Genetic analyses of diverse populations improves discovery for complex traits. *Nature* **570**, 514–518 (2019).

14. Sakaue, S. *et al.* A cross-population atlas of genetic associations for 220 human phenotypes. *Nat. Genet.* **53**, 1415–1424 (2021).

15. Yengo, L. *et al.* Meta-analysis of genome-wide association studies for height and body mass index in ~700000 individuals of European ancestry. *Hum. Mol. Genet.* **27**, 3641–3649 (2018).

16. Choi, S. W. & O'Reilly, P. F. PRSice-2: Polygenic Risk Score software for biobank-scale data. *GigaScience* **8**, giz082 (2019).

17. Ge, T., Chen, C.-Y., Ni, Y., Feng, Y.-C. A. & Smoller, J. W. Polygenic prediction via Bayesian regression and continuous shrinkage priors. *Nat. Commun.* **10**, 1776 (2019).

18. Ruan, Y. *et al.* Improving polygenic prediction in ancestrally diverse populations. *Nat. Genet.* **54**, 573–580 (2022).

19. Privé, F., Arbel, J. & Vilhjálmsson, B. J. LDpred2: better, faster, stronger. *Bioinformatics* **36**, 5424–5431 (2021).

20. Privé, F., Arbel, J., Aschard, H. & Vilhjálmsson, B. J. Identifying and correcting for misspecifications in GWAS summary statistics and polygenic scores. *Hum. Genet. Genomics Adv.* **3**, 100136 (2022).
