# Supplementary material for: Clinical utility of polygenic scores for cardiometabolic disease in Arabs
Source: Nat Commun. 2023 Oct 18;14:6535. doi: 10.1038/s41467-023-41985-1 (PMC10584889; doi:10.1038/s41467-023-41985-1)
Supplement: Supplementary file 3 — Reporting Summary [file 41467_2023_41985_MOESM3_ESM.pdf]

nature portfolio

Corresponding author(s): Akl C. Fahed, MD, MPH

Last updated by author(s): Sep 20, 2023

Reporting Summary

Nature Portfolio wishes to improve the reproducibility of the work that we publish. This form provides structure for consistency and transparency in reporting. For further information on Nature Portfolio policies, see our Editorial Policies and the Editorial Policy Checklist.

Statistics

For all statistical analyses, confirm that the following items are present in the figure legend, table legend, main text, or Methods section.

n/a	Confirmed	
☐	☒	The exact sample size (*n*) for each experimental group/condition, given as a discrete number and unit of measurement
☐	☒	A statement on whether measurements were taken from distinct samples or whether the same sample was measured repeatedly
☐	☒	The statistical test(s) used AND whether they are one- or two-sided<br>*Only common tests should be described solely by name; describe more complex techniques in the Methods section.*
☐	☒	A description of all covariates tested
☐	☒	A description of any assumptions or corrections, such as tests of normality and adjustment for multiple comparisons
☐	☒	A full description of the statistical parameters including central tendency (e.g. means) or other basic estimates (e.g. regression coefficient) AND variation (e.g. standard deviation) or associated estimates of uncertainty (e.g. confidence intervals)
☐	☒	For null hypothesis testing, the test statistic (e.g. *F*, *t*, *r*) with confidence intervals, effect sizes, degrees of freedom and *P* value noted<br>*Give P values as exact values whenever suitable.*
☐	☒	For Bayesian analysis, information on the choice of priors and Markov chain Monte Carlo settings
☐	☒	For hierarchical and complex designs, identification of the appropriate level for tests and full reporting of outcomes
☐	☒	Estimates of effect sizes (e.g. Cohen's *d*, Pearson's *r*), indicating how they were calculated

Our web collection on statistics for biologists contains articles on many of the points above.

Software and code

Policy information about availability of computer code

Data collection	The study population included a disease cohort of 5,399 individuals referred for cardiology care at the King Faisal Specialist Hospital and Research Center – a tertiary care hospital in Riyadh – from all five regions of Saudi Arabia, and a population reference cohort of 1,017 individuals not known to have cardiometabolic disease and that are representative of 28 tribes representative of indigenous Arabs in Saudi Arabia. Blood samples were collected for genetic data analysis. Phenotypic variables were obtained from chart abstraction using data available in the context of clinical care.
Data analysis	We used following software for analyses in this publication: R (version 4.0.0) for statistical analysis, PLINK (version 2.0) for genetic data analysis, KING (version 2.2.7) for kinship inference, FlashPCA (version 2.0) for principal components analysis, and PRSices-2 (version 2.3.5), LDpred2 (implemented under R package "bigsnpr" version 1.8.1), lassosum2 (implemented under R package "bigsnpr" version 1.8.1), PRS-CS (version 1.0.0), and PRS-CSx (version 1.0.0) for polygenic score calculation.

For manuscripts utilizing custom algorithms or software that are central to the research but not yet described in published literature, software must be made available to editors and reviewers. We strongly encourage code deposition in a community repository (e.g. GitHub). See the Nature Portfolio guidelines for submitting code & software for further information.

Data

Policy information about availability of data

All manuscripts must include a data availability statement. This statement should provide the following information, where applicable:

- Accession codes, unique identifiers, or web links for publicly available datasets
- A description of any restrictions on data availability
- For clinical datasets or third party data, please ensure that the statement adheres to our policy

Due to local privacy laws and privileged human information, all requests for raw genotyping and clinical data are subject to prior approval from the local IRB. For the raw data for Arab cohort, the local IRB can be reached at ORA@kfshrc.edu.sa with an expected timeframe for response of two months. Analysis of the UK Biobank data was performed using application 31224 and approved by King Abdullah University of Science and Technology (KAUST) IRB. The UK Biobank data are available to researchers with research inquiries following IRB and UK Biobank approval (https://www.ukbiobank.ac.uk/enable-your-research/apply-for-access). The GWAS Catalog (https://www.ebi.ac.uk/gwas/downloads/summary-statistics) contains all GWAS summary statistics. Ancestry-matched LD reference panels built with 1000 Genomes Project phase 3 samples are available at https://github.com/getian107/PRScsx. The polygenic scores described in this publication are available for download from the Polygenic Score Catalog (https://www.pgscatalog.org) under the publication ID PGP000501 and the score IDs PGS003866-PGS003891.

Research involving human participants, their data, or biological material

Policy information about studies with human participants or human data. See also policy information about sex, gender (identity/presentation), and sexual orientation and race, ethnicity and racism.

Reporting on sex and gender	In this study, we considered only the biological sex. Sex is self-reported data, and samples with a mismatch between genotypically-determined and self-reported sex were removed in the process of genetic data quality control.
Reporting on race, ethnicity, or other socially relevant groupings	This manuscript is specifically focused on studying Arabs, a heterogeneous groups that is socially and genetically diverse, which we define in the introduction of the manuscript. We also discuss indigenous Arabs from Saudi Arabia and report principal components of ancestry comparison between this group and the 1000 genomes continental ancestry groups. Finally, we use a matching technique based on genetic distance to define a small group in the UK Biobank that is genetically matched to the indigenous Arabs form Saudi Arabia. We define this group as "Arab-matched" and report on the self-reported race and ancestry of individuals in this group.
Population characteristics	In a case-control cohort of 5,399 participants, 64.3% are male and the mean age is 54.83 years (standard deviation 14.82). The outcomes were three cardiometabolic diseases diagnosed and treated in the past and seven continuous measurements. Lipid levels and blood pressures were adjusted by medication use. Adjustments were made to regression models for age, sex, array version, and the first 10 principal components of ancestry.
Recruitment	The study population included a disease cohort of 5,399 individuals referred for cardiology care at the King Faisal Specialist Hospital and Research Center – a tertiary care hospital in Riyadh – from all five regions of Saudi Arabia, and a population reference cohort of 1,017 individuals not known to have cardiometabolic disease and that are representative of 28 tribes representative of indigenous Arabs in Saudi Arabia. Participants were approached in clinic by research coordinators for consent and participation in the study.
Ethics oversight	The study was approved by the institutional review board of the King Faisal Specialist Hospital and Research Center (KFSHRC RAC# 2190011).

Note that full information on the approval of the study protocol must also be provided in the manuscript.

Field-specific reporting

Please select the one below that is the best fit for your research. If you are not sure, read the appropriate sections before making your selection.

☒ Life sciences ☐ Behavioural & social sciences ☐ Ecological, evolutionary & environmental sciences

For a reference copy of the document with all sections, see nature.com/documents/nr-reporting-summary-flat.pdf

Life sciences study design

All studies must disclose on these points even when the disclosure is negative.

Sample size	We note that there is worldwide scarcity of Arab cohorts available for genetic studies. No sample size calculation was performed. This was convenience sampling of participants referred to care. The sample size of ~6500 participants with measurement of continuous traits and with large prevalence of binary traits exceeded many similar studies of establishing association of polygenic score with a trait and allowed randomly splitting the sample into a validation and testing datasets while still establishing strong associations. The sample size was sufficient as it exceeded the samples sizes of many similar studies demonstrating strong associations of polygenic score with traits/disease in smaller sample sizes.
Data exclusions	Out of 6,566 samples, quality control excluded 150 samples due to variant calling missingness > 5%, heterozygosity rate > 5 standard deviations above the mean, and mismatch between genotypically-determined and self-reported sex, resulting in 6,416 samples consisting of 5,399 patients and 1017 reference samples.

Replication	We followed two approaches to replicate the findings of this study. First, we followed a design that allows an internal replication through finding an optimal polygenic score in a validation set then testing the best score in a separate testing dataset. Second, we established an Arab-matched cohort in the UK Biobank in which we also reported the performance of the optimized polygenic scored derived in Arabs. Replication attempts were successful.
Randomization	In this case-control study, we randomly divided samples into validation (N=2,700) and testing (N=2,699) datasets in order to determine the optimal polygenic score model.
Blinding	Blinding was not relevant to this observational study.

Reporting for specific materials, systems and methods

We require information from authors about some types of materials, experimental systems and methods used in many studies. Here, indicate whether each material, system or method listed is relevant to your study. If you are not sure if a list item applies to your research, read the appropriate section before selecting a response.

Materials & experimental systems

n/a	Involved in the study
☒	☐ Antibodies
☒	☐ Eukaryotic cell lines
☒	☐ Palaeontology and archaeology
☒	☐ Animals and other organisms
☒	☐ Clinical data
☒	☐ Dual use research of concern
☒	☐ Plants

Methods

n/a	Involved in the study
☒	☐ ChIP-seq
☒	☐ Flow cytometry
☒	☐ MRI-based neuroimaging
